# Partial Covariance-Based Detectors for Cooperative Spectrum Sensing in Cognitive Communications

**DOI:** 10.3390/s26082557

**Published:** 2026-04-21

**Authors:** Dayan Adionel Guimarães

**Affiliations:** National Institute of Telecommunications (Inatel), Av. João de Camargo, 510, Santa Rita do Sapucaí 37536-001, MG, Brazil; dayan@inatel.br; Tel.: +55-35-3471-9227

**Keywords:** cognitive radio, covariance-based detectors, dynamic spectrum access, partial sample covariance matrix, spectrum sensing

## Abstract

This article proposes modified test statistics for six blind covariance-based detectors used in data fusion cooperative spectrum sensing, where the full Hermitian sample covariance matrix (SCM) of the received signal is replaced by a symmetric real-valued partial sample covariance matrix (PSCM). This substitution results in a substantial reduction in overall computational complexity compared to the original SCM-based formulations, while preserving or improving detection accuracy under realistic conditions that include non-uniform noise powers, time-varying distance-dependent path loss, spatially correlated shadowing, and multipath fading with a random Rice factor. The computation of the PSCM requires 50% fewer floating-point operations than the full SCM and offers a hardware-friendly structure due to its reliance on real-valued arithmetic. On the test statistic side, the adoption of the PSCM leads to computational costs ranging from 3.37% to 61.9% of those incurred by the corresponding SCM-based test statistics.

## 1. Introduction

The rapid expansion of wireless communication systems in recent years has contributed to an increasing scarcity of the radio-frequency (RF) spectrum. This situation primarily arises from the prevalent fixed spectrum allocation policy, which assigns exclusive usage rights to the primary user (PU) network. However, despite this allocation, the designated frequency bands may remain underutilized in specific geographic areas or during particular time periods, ultimately leading to inefficient exploitation of the available spectrum resources.

The large-scale deployment of the Internet of Things (IoT) and fifth-generation (5G) wireless communication networks is expected to further exacerbate the RF spectrum shortage. This is because the substantial number of expected terminals will require significantly higher bandwidths, a tendency that is also projected to intensify in beyond-5G network scenarios. To mitigate this scarcity, cognitive radio (CR) networks can be employed to exploit unoccupied frequency bands through dynamic spectrum access policies, wherein cognitive secondary user (SU) terminals opportunistically utilize the available vacant bands.

Spectrum sensing [1,2,3], whether performed independently or supported by an RF spectrum occupancy database [4], enables the secondary network to identify spectral holes. Nonetheless, it is well established that independent spectrum sensing conducted by each SU individually is susceptible to performance degradation, which has motivated the adoption of cooperative spectrum sensing (CSS) as the preferred approach. In CSS, multiple SUs collaborate in the sensing process to enhance the reliability and accuracy of decisions regarding the channel occupancy state. The most prevalent form of CSS is the centralized scheme, wherein the sensing information collected by the SUs is transmitted to a fusion center (FC) within the secondary network, which then determines the state of the monitored channel.

Spectrum sensing can be formulated as a binary hypothesis testing problem, in which the secondary user aims to decide between the null hypothesis, $H_{0}$, corresponding to the absence of the PU signal, and the alternative hypothesis, $H_{1}$, indicating its presence. In this framework, a test statistic is computed from the received signal samples and compared against a predefined decision threshold to infer the true state of the channel. The effectiveness of the sensing procedure is commonly quantified by the probability of detection, $P_{d}$, which measures the likelihood of correctly identifying the presence of the PU signal, and the probability of false alarm, $P_{\mathrm{fa}}$, which represents the chance of erroneously declaring the channel as occupied when it is actually idle. An appropriate trade-off between these probabilities must be achieved to ensure both reliable spectrum utilization and minimal interference to the primary network.

### 1.1. Related Work

The detectors employed for spectrum sensing can be broadly classified into two main categories: blind and non-blind. Blind detectors do not require any prior knowledge about the PU signal, the characteristics of the sensing channel, or the noise parameters, whereas non-blind detectors rely on one or more of these pieces of information.

Representative examples of blind detectors include the covariance absolute value (CAV) detector [5], the Gini index detector (GID) [6], the Gerschgorin radii and centers ratio (GRCR) detector [7], the Pietra–Ricci index detector (PRIDe) [8], the Theil index detector (TID) [9], the Atkinson index detector (AID) [10], the Hadamard ratio (HR) detector [11], the volume-based detector number 1 (VD1) [12], the scaled largest eigenvalue (SLE) [13], the maximum–minimum eigenvalue detector (MMED) [14], the arithmetic-to-geometric mean (AGM) detector [15], the locally most powerful invariant test (LMPIT) [16], and the mean-to-square extreme eigenvalue (MSEE) detector [17]. The test statistics associated with these detectors are derived through operations applied to the sample covariance matrix (SCM) of the received signal.

In contrast, typical examples of non-blind detectors are the well-known energy detector (ED) [18], the cyclostationary feature detector (CFD) [19], the maximum eigenvalue detector (MED) [14], and the matched filter (MF) [20]. While the ED and MED do not necessitate prior information about the PU signal, they do require knowledge of the noise variance. Conversely, the CFD and MF depend on specific information about the received PU signal.

This study focuses exclusively on blind detectors, since in practical scenarios it is common for only limited information about the primary signal and sensing channel to be available, and any information about the noise statistics is often incomplete or unreliable. Among the various blind detectors, particular attention is given to those whose test statistics are constructed directly from the elements of the SCM, without resorting to eigenvalue decomposition, eigenvectors, determinants, or other computationally demanding operations. Specifically, this work concentrates on the detectors GID, GRCR, PRIDe, TID, AID, and LMPIT.

### 1.2. Contributions and Organization of the Article

This article proposes reformulated test statistics for the GID, GRCR, PRIDe, TID, AID, and LMPIT detectors. These alternative formulations are derived by employing the elements of the so-called partial sample covariance matrix (PSCM) instead of the full SCM.

Given the complex matrix Y containing the signal samples received by the SUs, it is well established that the positive definite Hermitian SCM can be expressed as $R=YY^{†}$, where † denotes the conjugate transpose operation. In contrast, the PSCM is a real symmetric matrix defined by $S=ℜ\left(Y\right)ℜ{\left(Y\right)}^{T}+ℑ\left(Y\right)ℑ{\left(Y\right)}^{T}$, where T represents the transposition, and ℜ(Y) and ℑ(Y) are the matrices containing the real and imaginary parts of Y, respectively.

The resulting simplified detectors achieve significant reductions in computational complexity while maintaining performance comparable to, and in certain scenarios surpassing, that of the original SCM-based counterparts.

As a byproduct of this study, the comprehensive MATLAB R2024a code [21] is made available to evaluate the performance of all detectors, thereby enabling reproducibility of the numerical results as well as the generation of additional ones.

The remainder of this article is organized as follows. Section 2 presents the system models. Section 3 reviews the original SCM-based test statistics. Section 4 introduces the PSCM concept. Section 5 presents the proposed PSCM-based test statistics. Section 6 provides the complexity analysis. Section 7 reports the numerical results. Section 8 concludes the article. The expected values required in the analysis are derived in Appendices Appendix A, Appendix B and Appendix C, while the detailed FLOP count analysis is presented in Appendix D.

## 2. System Models

This section is devoted to the modeling of the transmitted and received signals, the noise, the sensing channel, and the spectrum sensing process itself. To the best of the author’s knowledge, the system model adopted herein constitutes the most realistic framework employed in the study of spectrum sensing, thereby enhancing the credibility of the results and analyses. Particular attention is given to the sensing channel model, which accounts for distance-dependent received signal strengths, spatially correlated shadowing, non-uniform noise power levels, and multipath fading characterized by an environment-dependent random Rice factor. Additionally, a novel signal-to-noise ratio (SNR) calibration procedure is incorporated to further improve the accuracy and applicability of the results.

### 2.1. Signal, Noise and Channel Models

The secondary network is composed of *m* SUs uniformly distributed within a circular coverage region of radius *r* centered in (0,0), along with a fixed single PU transmitter located at configurable coordinates (x,y). For each sensing round, the positions of the SUs are re-sampled to emulate node mobility. Each SU acquires *n* samples of the PU signal during the designated sensing interval. The samples collected by the *m* SUs are transmitted to the FC, where the matrix $Y\in C^{m\times n}$ is constructed as

(1) $$Y=hx^{T}+V,$$

where $h\in C^{m\times 1}$ denotes the channel vector, $x\in C^{n\times 1}$ contains the samples of the transmitted PU signal, and $V\in C^{m\times n}$ represents the Gaussian noise matrix.

The PU signal vector x in (1) represents a baseband quaternary phase-shift keying (QPSK) signal with $N_{s}$ samples per symbol. The sensing channel is characterized by the vector h, whose elements $h_{i}$, for i=1,…,m, denote the channel gains between the PU transmitter and the *i*th SU. These gains are assumed to remain constant over the sensing interval and to be independent and identically distributed across different sensing events. The assumption of constant gains reflects a flat fading [22] channel, while the independence across sensing events models the scenario in which the time between consecutive sensing rounds exceeds the channel coherence time [22]. The channel vector is computed as

(2) h=Ga,

where $a\in C^{m\times 1}$ is a vector of complex Gaussian random variables, whose elements $a_{i}$ have mean $E\left[a_{i}\right]=\mu_{ℜ\left(a_{i}\right)}+j\mu_{ℑ\left(a_{i}\right)}=\sqrt{K_{i}/\left(K_{i}+1\right)}+j0$ and variance $\mathrm{var}\left[a_{i}\right]=\mathrm{var}\left[ℜ\left(a_{i}\right)\right]+\mathrm{var}\left[ℑ\left(a_{i}\right)\right]=1/\left(K_{i}+1\right)$, where $K_{i}=E^{2}\left[a_{i}\right]/\mathrm{var}\left[a_{i}\right]=10^{K_{i}^{\mathrm{dB}}/10}$, with $K_{i}^{\mathrm{dB}}=10\log_{10}\left(K_{i}\right)$ denoting the Rice factor in dB for the channel linking the PU transmitter to the *i*th SU. According to [23], $K_{i}$ can be modeled as a Gaussian random variable (in dB) with mean $\mu_{K}$ and standard deviation $\sigma_{K}$, which are determined by the propagation characteristics of the environment. For example, in urban environments, typical values are $\mu_{K}=1.88$ dB and $\sigma_{K}=4.13$ dB, whereas suburban areas are associated with $\mu_{K}=2.41$ dB and $\sigma_{K}=3.84$ dB. In rural or open settings, $\mu_{K}=2.63$ dB and $\sigma_{K}=3.82$ dB [23]. In this study, an urban scenario has been adopted as the default reference case.

Non-uniform received signal powers across the SUs are considered, arising from the combined effects of signal shadowing and the varying distances between the PU transmitter and the SUs. This variability is captured by the diagonal gain matrix $G\in R^{m\times m}$ present in (2), which is given by

(3) $$G=\mathrm{diag}\left(\sqrt{\frac{p}{P_{\mathrm{tx}}}}\right),$$

where $p={\left[{P_{\mathrm{rx}}}_{1}\;{P_{\mathrm{rx}}}_{2}\;\ldots \;{P_{\mathrm{rx}}}_{m}\right]}^{T}$ denotes the vector containing the received PU signal powers at the *m* SUs, $P_{\mathrm{tx}}$ is the transmitted PU signal power, and diag(·) denotes the operation that forms a diagonal matrix whose diagonal elements are specified by the argument vector.

The local-mean power of the signal received by the *i*th SU is determined according to the log-distance path loss model discussed in [22]. In dBm (i.e., dB relative to 1 milliwatt), this received signal power is

(4) $$P_{{\mathrm{rx}}_{i}}^{\mathrm{dBm}}=10\log_{10}\left[10^{3}P_{\mathrm{tx}}{\left(\frac{d_{0}}{d_{i}}\right)}^{\eta }\right]+S_{i},$$

where $d_{0}$ is a reference distance in the far-field region of the PU transmit antenna, $d_{i}$ is the distance from the PU transmitter to the *i*th SU, η is the dimensionless, environment-dependent path loss exponent, and $S_{i}$ models the log-normal signal shadowing component affecting the *i*th SU, represented as a zero-mean Gaussian random variable in dB with a standard deviation $\sigma_{s}$. Typical values of $\sigma_{s}$ can be found, for instance, in [24], according to the morphology of the environment.

If the local-mean received signal power is expressed in watts, it follows that

(5) $${P_{\mathrm{rx}}}_{i}=10^{-3}10^{P_{{\mathrm{rx}}_{i}}^{\mathrm{dBm}}/10}.$$

A specific value of $S_{i}$ in (4) represents the realization of a location-dependent random variable that corresponds to an element of the Gaussian random matrix $S_{c}$. The realizations of $S_{i}$ over a square area with side length 2r, within which the circular SU coverage region is inscribed, collectively compose the full matrix $S_{c}$, as further described in the following.

The row-wise and column-wise correlations among the elements of $S_{c}$ are determined by the covariance matrix Σ, which is constructed based on the well-known negative-exponential correlation model. This model is defined by the correlation function R(δ)=exp(−δ/Λ), where δ denotes the distance between two points, and Λ (ranging from $0^{+}$ to the number of rows of Σ) specifies the correlation length, defined as the distance at which the shadowing correlation coefficient decreases to 1/e. Accordingly, the elements of Σ are expressed as $\Sigma_{z,w}=\exp\left(-\delta_{z,w}/\Lambda \right)$, where $\delta_{z,w}$, which takes values as integer multiples of $\sqrt{2}$, represents the distance between the diagonal of Σ and the point indexed by row *z* and column *w* of this matrix.

Given a square matrix $S_{u}$ with uncorrelated normal random variables, the Gaussian matrix $S_{c}$ with correlated values (associated with the set of spatially correlated points) is formed through the transformation

(6) $$S_{c}=\sigma_{s}LS_{u}L^{T},$$

where L is the lower-triangular matrix obtained from the Cholesky decomposition of Σ.

Returning to the description of the model defined in (1), it is recognized that, in practice, the thermal noise levels generated at the receivers’ front-ends are not identical, mainly due to uncalibrated hardware and variations in ambient temperature. Moreover, this non-uniform noise may fluctuate over time, and unwanted interfering signals entering the receivers can further create the appearance of changing noise levels. In this work, to capture the non-uniformity of noise levels, the elements in the *i*th row of $V\in C^{m\times n}$ are modeled as independent Gaussian random variables with zero mean and variance

(7) $$\sigma_{i}^{2}=\left(1+\rho u\right){\bar{\sigma }}^{2},$$

where *u* denotes a realization of the random variable *U*, which is uniformly distributed over [−1,1], ${\bar{\sigma }}^{2}$ is the noise variance averaged across all SUs, and 0≤ρ<1 specifies the fractional variation of the noise power relative to the average ${\bar{\sigma }}^{2}$.

### 2.2. SNR Calibration

The SNR averaged across the SUs is defined as

(8) $$\mathrm{SNR}=\frac{1}{m}\sum_{i=1}^{m}E\left[\frac{{P_{\mathrm{rx}}}_{i}}{\sigma_{i}^{2}}\right].$$

where E[·] denotes the expectation operator. Because the received signal is independent of the noise, and both $P_{{\mathrm{rx}}_{i}}$ and $\sigma_{i}^{2}$ are identically distributed random variables with respect to the index *i*, owing to the uniform distribution of SUs within the coverage area, this index can be omitted, yielding

(9) $$\mathrm{SNR}=E\left[P_{\mathrm{rx}}\right]E\left[\frac{1}{\sigma^{2}}\right].$$

From (4), the equation for the total (area-mean distance-dependent plus local-mean-shadowed) signal power, in dBm, received by the *i*th SU can be written as

(10) $$P_{{\mathrm{rx}}_{i}}^{\mathrm{dBm}}=10\log_{10}\left[10^{3}P\left(d_{i}\right)\right]+S_{i},$$

where $P\left(d_{i}\right)=P_{\mathrm{tx}}{\left(d_{0}/d_{i}\right)}^{\eta }$ accounts solely for the distance-dependent part of the received signal power. In watts,

(11) $$P_{{\mathrm{rx}}_{i}}=P\left(d_{i}\right)10^{S_{i}/10}.$$

Since the area-mean power of the received signal is evaluated over a sufficiently large region to average out both the local-mean shadowing and the fast (instantaneous) fading effects, the distance-dependent component and the shadowed component of the received signal in (11) can be regarded as statistically independent. From another perspective, the well-established model (4) represents the received signal power as the sum of two independent terms: one accounting for the distance-dependent attenuation and the other for shadowing. Consequently, the expected value of the total received signal power across the SUs can be expressed as

(12) $$E\left[P_{\mathrm{rx}}\right]=E\left[P\left(d\right)\right]E\left[10^{S/10}\right],$$

where it has also been taken into account that $P_{{\mathrm{rx}}_{i}}$, $P(d_{i})$ and $S_{i}$, individually, are identically distributed random variables (due to uniform distribution of SUs and the same shadowing distribution throughout the coverage area).

The expected values E[P(d)] and $E[10^{S/10}]$ operated in (12) are derived in Appendix A and Appendix B, yielding

(13) $$\begin{array}{cc} E[P(d)] & =\frac{P_{\mathrm{tx}}}{\pi r^{2}d_{0}^{-\eta }}\int_{0}^{2\pi }\int_{0}^{r}\left[{\left(z\cos\theta -x_{\mathrm{PU}}\right)}^{2}\right.\\ \; & \;{\left.+{\left(z\sin\theta -y_{\mathrm{PU}}\right)}^{2}\right]}^{-\frac{\eta }{2}}z\;dz\;d\theta , \end{array}$$

and

(14) $$E\left[10^{S/10}\right]=\exp\left[\frac{\sigma^{2}\ln^{2}\left(10\right)}{200}\right].$$

The expected value of $1/\sigma^{2}$ calculated in (9) is derived in Appendix C. For 0<ρ<1, it follows that

(15) $$E\left[\frac{1}{\sigma^{2}}\right]=\frac{1}{2{\bar{\sigma }}^{2}\rho }\ln\left(\frac{1+\rho }{1-\rho }\right).$$

For ρ=0, the expected value of $1/\sigma^{2}$ becomes

(16) $$E\left[\frac{1}{\sigma^{2}}\right]=\frac{1}{{\bar{\sigma }}^{2}}.$$

The result (15) or (16), plus the result (12) obtained from (13) and (14), is substituted into (9), yielding

(17) $$\mathrm{SNR}=E\left[P(d)\right]E\left[10^{S/10}\right]E\left[\frac{1}{\sigma^{2}}\right],$$

from which ${\bar{\sigma }}^{2}$ can be retrieved for a given predefined SNR, since $E[1/\sigma^{2}]$ is a function of ${\bar{\sigma }}^{2}$.

In the final stage of the modeling process, ${\bar{\sigma }}^{2}$ is substituted into (7) together with the corresponding realizations of the random variable *U*, resulting in the variance $\sigma_{i}^{2}$ of the noise samples in the *i*th row of the matrix V. For the purpose of computer simulations, new values for the set $\sigma_{i}^{2}$ are generated for each sensing event, giving a space-time varying characteristic to the noise power.

The matrix Y given in (1) is subsequently computed at the FC. Under the hypothesis that there is a vacant spectrum band, Y=V. Otherwise, $Y=hx^{T}+V$.

## 3. Original SCM-Based Test Statistics

This section presents the original test statistics of the detectors PRIDe, GIG, GRCR, AID, TID and LMPIT, which are built from the elements of the full SCM.

Let $Y\in C^{m\times n}$ be the data matrix corresponding to the samples of the PU signal processed by the FC. Define the SCM (without division by *n*)

(18) $$R=YY^{†}\in C^{m\times m},$$

with elements $R_{ij}$ for i,j=1,…m, and define

r=vec(R)

as the vectorized version of R, where $r_{i}$, $i=1,\ldots ,m^{2}$ is the *i*th element of r, and let the mean of the elements in r be

$$\bar{r}=\frac{1}{m^{2}}\sum_{i=1}^{m^{2}}r_{i}.$$

The test statistics for the detectors PRIDe [8], GID [6], GRCR [7], AID [10], TID [9], and LMPIT [16] are respectively given by

(19) $$T_{\mathrm{PRIDe}}=\frac{\sum_{i=1}^{m^{2}}\left|r_{i}\right|}{\sum_{i=1}^{m^{2}}\left|r_{i}-\bar{r}\right|},$$

(20) $$T_{\mathrm{GID}}=\frac{2\left(m^{2}-m\right)\sum_{i=1}^{m^{2}}\left|r_{i}\right|}{\sum_{u=1}^{m^{2}}\sum_{j=1}^{m^{2}}\left|r_{u}-r_{j}\right|},$$

(21) $$T_{\mathrm{GRCR}}=\frac{\sum_{i=1}^{m}\left(\sum_{j=1}^{m}\left|R_{ij}\right|-R_{ii}\right)}{\sum_{i=1}^{m}R_{ii}},$$

(22) $$T_{\mathrm{AID}}=\frac{{\left(\sum_{i=1}^{m}\sum_{j=1}^{m}R_{ij}^{1-\epsilon }\right)}^{\frac{1}{1-\epsilon }}}{\sum_{i=1}^{m}\sum_{j=1}^{m}R_{ij}},$$

(23) $$T_{\mathrm{TID}}=\frac{\mu }{\sum_{i=1}^{m}\sum_{j=i}^{m}\left(2-\delta_{ij}\right)\;\left|R_{ij}\right|\ln\left(\frac{|R_{ij}|}{\mu }\right)},$$

(24) $$T_{\mathrm{LMPIT}}=\sum_{i=1}^{m}\sum_{j=1}^{m}{\left|C_{ij}\right|}^{2},$$

where 0<ϵ<1 is the inequality aversion parameter of the Atkinson index, $\mu =\frac{1}{m^{2}}\sum_{i=1}^{m}\sum_{j=1}^{m}\left|R_{ij}\right|$, $\delta_{ij}$ is the Kronecker delta function ($\delta_{ij}=1$ if i=j and $\delta_{ij}=0$ if i≠j), $C=D^{-1/2}RD^{-1/2}$, and D=diag(R).

It is worth noticing that the GRCR test statistic (21) can be rewritten as

$$\begin{array}{cc} T_{\mathrm{GRCR}} & =\frac{\sum_{i=1}^{m}\sum_{j=1}^{m}\left|R_{ij}\right|-\sum_{i=1}^{m}R_{ii}}{\sum_{i=1}^{m}R_{ii}}\\ \; & =\frac{\sum_{i=1}^{m}\sum_{j=1}^{m}\left|R_{ij}\right|}{\sum_{i=1}^{m}R_{ii}}-1\equiv \frac{\sum_{i=1}^{m}\sum_{j=1}^{m}\left|R_{ij}\right|}{\sum_{i=1}^{m}\left|R_{ii}\right|}, \end{array}$$

which is the CAV test statistic proposed in [5], since $R_{ii}=\left|R_{ii}\right|$. Thus, the GRCR and CAV are equivalent detectors.

Additionally it is worth noting that $T_{\mathrm{AID}}$ is real-valued, which is explained as follows. The SCM R is Hermitian, which implies $R_{ji}={R_{ij}}^{*}$. As a result, all off-diagonal elements appear in complex-conjugate pairs. When summed, each pair contributes a real value, since $R_{ij}+R_{ji}=2ℜ\left\{R_{ij}\right\}$. The diagonal elements are real by definition. This conjugate symmetry is preserved after applying the real exponent 1−ϵ, so the sum $\sum_{i,j}R_{ij}^{1-\epsilon }$ remains real. The denominator of $T_{\mathrm{AID}}$, being a sum of $\left\{R_{ij}\right\}$, is also real. Therefore, $T_{\mathrm{AID}}$, which is formed from real quantities through real operations, is itself real-valued.

## 4. The Partial Sample Covariance Matrix

The full sample covariance matrix, R, defined in (18) can be written as

(25) R=S+jT,

where the m×m real symmetric matrix

(26) $$S=ℜ\left(Y\right)ℜ{\left(Y\right)}^{T}+ℑ\left(Y\right)ℑ{\left(Y\right)}^{T}$$

is called herein the partial sample covariance matrix (PSCM), with elements $S_{ij}$ for i,j=1,…m, and $T=ℑ\left(Y\right)ℜ{\left(Y\right)}^{T}-ℜ\left(Y\right)ℑ{\left(Y\right)}^{T}$ is also a real symmetric matrix. The vectorized version of S is

s=vec(S),

and the corresponding mean is

(27) $$\bar{s}=\frac{1}{m^{2}}\sum_{i=1}^{m^{2}}s_{i},$$

with $s_{i}$, $i=1,\ldots ,m^{2}$, being the *i*th element of s.

The adoption of the PSCM, S, in place of the full Hermitian SCM, R, can be justified from complementary statistical, algorithmic, and practical implementation perspectives. The following subsections address these topics.

### 4.1. Preservation of Sufficient Statistical Information

From the definitions (25) and (26) it is clear that the PSCM corresponds exactly to the real part of the SCM; that is, S=ℜ(R). Consequently, S preserves the energy and correlation information contained in R, while discarding only the skew-symmetric imaginary component associated with cross-quadrature interactions.

Under the null hypothesis $H_{0}$, where Y=V and the noise samples are circularly symmetric complex Gaussian, the expected value of the imaginary part of R is zero. Hence, S constitutes a sufficient second-order statistic under $H_{0}$, and its statistical behavior remains fully consistent with that of R in noise-only conditions.

Under the alternative hypothesis $H_{1}$, the dominant contribution to the SCM arises from the rank-one signal term $hx^{T}$, whose impact on the covariance structure is primarily captured by the real-valued quadratic forms embedded in S. Therefore, the discriminatory power between $H_{0}$ and $H_{1}$ is preserved when R is replaced by S.

### 4.2. Robustness to Noise Uncertainty and Non-Idealities

Several of the detectors investigated in this work rely on global inequality measures, sums of magnitudes, or normalized correlations between covariance entries, rather than on phase-sensitive quantities. In such cases, the imaginary part of R often contributes mainly to variance inflation rather than to useful discriminatory information.

By suppressing the imaginary component, the PSCM reduces sensitivity to random phase fluctuations, particularly under scenarios involving non-uniform noise powers, spatially correlated shadowing, and time-varying multipath fading.

Moreover, since the PSCM depends exclusively on real-valued operations, the resulting test statistics preserve the constant false alarm rate (CFAR) property, as confirmed numerically in Section Constant False Alarm Rate. The invariance of decision thresholds with respect to noise variance remains unaffected by the substitution of R with S.

### 4.3. Computational and Hardware Efficiency

From a computational standpoint, the PSCM offers a substantial reduction in arithmetic complexity. When exploiting symmetry, the computation of S requires approximately half the number of floating-point operations needed to compute R, as detailed in Appendix D. This reduction is not limited to the covariance estimation stage, but propagates to the test statistics themselves due to the elimination of complex arithmetic and absolute-value operations.

From a hardware implementation perspective, the PSCM is particularly attractive, since it relies solely on real-valued multiplications and additions. This property enables more efficient fixed-point implementations, reduced memory bandwidth, and simpler datapath architectures when compared to full complex-valued SCM processing.

### 4.4. Structural Compatibility with Covariance-Based Detectors

The detectors addressed in this work, namely PRIDe, GID, GRCR, AID, TID, and LMPIT, are fundamentally constructed from element-wise operations on covariance entries, such as sums, absolute differences, ratios, and normalized correlations. None of these detectors explicitly exploits phase coherence or eigenstructure information, which further supports the suitability of using S instead of R.

In fact, the symmetry and real-valued nature of S enable additional simplifications in the formulation of the test statistics, as shown in Section 5, without altering their conceptual foundations. These simplifications are not arbitrary, but emerge naturally from the structural properties of the PSCM.

## 5. Proposed PSCM-Based Test Statistics

The proposed simplified PRIDe (sPRIDe) test statistic is

(28) $$T_{\mathrm{sPRIDe}}=\frac{\sum_{i=1}^{m^{2}}s_{i}}{\sum_{i=1}^{m^{2}}\left|s_{i}-\bar{s}\right|},$$

where the elements of S replace the elements of R adopted in (19). Additionally, the absolute values adopted in the numerator of (19) have been removed in (28).

The simplified GID (sGID) test statistic proposed herein is

(29) $$T_{\mathrm{sGID}}=\frac{\sum_{i=1}^{m^{2}}s_{i}}{\sum_{u=1}^{m^{2}}\sum_{j=u}^{m^{2}}\left|s_{u}-s_{j}\right|}.$$

Notice that, besides operating on the elements of the real matrix S instead of R, the test statistic $T_{\mathrm{sGID}}$ sums fewer terms in the denominator due to the symmetry of S. The absolute values adopted in the numerator of (20) have been removed in (29). Moreover, the multiplying constant present in (20) has been removed in (29), as it does not affect performance.

The proposed simplified GRCR (sGRCR) test statistic also adopts S instead of R, achieving further simplifications due to the removal of the absolute value operation present in (21); that is,

$$\begin{array}{cc} T_{\mathrm{sGRCR}} & =\frac{\sum_{i=1}^{m}\sum_{j=1}^{m}S_{ij}-\sum_{i=1}^{m}S_{ii}}{\sum_{i=1}^{m}S_{ii}}\\ \; & =\frac{\sum_{i=1}^{m}\sum_{j=1}^{m}S_{ij}}{\sum_{i=1}^{m}S_{ii}}-1, \end{array}$$

from where the subtraction of 1 can be removed, yielding a test statistic that is the ratio between the sum of all elements of S and its trace. However, in terms of equivalence, the test statistic can be further simplified by modifying the numerator to sum only the upper triangular elements of S. The final sGRCR test statistic then becomes

(30) $$T_{\mathrm{sGRCR}}=\frac{\sum_{i=1}^{m}\sum_{j=i+1}^{m}S_{ij}}{\sum_{i=1}^{m}S_{ii}}.$$

The simplified AID (sAID) test statistic is the one that attains the largest degree of simplification with respect to the original $T_{\mathrm{AID}}$ given in (22). When S is used in place of R in (22), negative entries of S may lead to complex-valued results due to the fractional exponent. To avoid this issue, only the positive elements of S are retained in the computation of $T_{\mathrm{sAID}}$. Although this modification may, in principle, discard part of the available information, simulation results indicate that the resulting detector exhibits robust performance and reduced sensitivity to the inequality aversion parameter ϵ. Consequently, ϵ=0.5 has been adopted, yielding the $T_{\mathrm{sAID}}$ test.

(31) $$T_{\mathrm{sAID}}=\frac{{\left(\sum_{j=1}^{J}\sqrt{z_{j}}\right)}^{2}}{\sum_{j=1}^{J}z_{j}},$$

where $z_{j}$ is the *j*th element of the vector z formed by the positive elements of S, and *J* is the cardinality of z. There are $(m^{2}-m)/2$ positive elements of S, on average; that is, $E\left[J\right]=\left(m^{2}-m\right)/2$, which translates into an important reduction in the computational cost.

The simplified TID (sTID) test statistic proposed herein is

(32) $$T_{\mathrm{sTID}}=\frac{\bar{s}}{\sum_{i=1}^{m}\sum_{j=i}^{m}\left(2-\delta_{ij}\right)\;S_{ij}\ln\left(\frac{|S_{ij}|}{\bar{s}}\right)},$$

where $\bar{s}$ is defined in (27), and $\delta_{ij}$ is the Kronecker delta function. The sTID test statistic adopts S instead of R, and removes the absolute value from the computation of $\bar{s}$ and outside the natural logarithm with respect to (23).

The proposed simplified LMPIT (sLMPIT) test statistic is

(33) $$T_{\mathrm{sLMPIT}}=\sum_{i=1}^{m}\sum_{j=1}^{m}C_{ij}^{2}={∥C∥}_{F}^{2},$$

where $C=D^{-1/2}SD^{-1/2}$, D=diag(S), and ${∥C∥}_{F}$ is the Frobenius norm of C. Besides using S instead of R, the single additional modification of $T_{\mathrm{sLMPIT}}$ with respect to $T_{\mathrm{LMPIT}}$ is the removal of the absolute value operation on the elements of C.

## 6. Complexity Analysis

Appendix D presents a detailed computational complexity analysis of the test statistics considered in this study. This complexity is evaluated in terms of the number of floating-point operations (FLOPs), which estimates the algorithmic workload independently of the execution environment. Time complexity is also evaluated as a complementary metric in Section 7, through measured runtime in MATLAB. The runtime reflects the actual execution time including overheads, memory access, and software-level optimizations. It is important to highlight that the measured runtime in MATLAB may not follow the same growth pattern of the theoretical computational complexity in FLOPs.

The computational complexities are summarized in Table 1, from where it can be seen that, except in the case of GID/sGID, whose dominant operations reside in the test statistic computation, the dominant operations for all remaining test statistics refer to the computation of the matrices R or S. Moreover, it is clear the important complexity reduction achieved by all simplified detectors with respect to the original ones.

The rightmost column of Table 1 shows, for each detector type, the ratio between the FLOP count of the simplified PSCM-based variant and the FLOP count of the original SCM-based approach, for m=10 and n=100. It can be seen, for example, that although the GID/sGID require the largest numbers of FLOPs, the sGID needs around 1/3 of the GID’s FLOP count. The smallest FLOP count has been attained by the GRCR/sGRCR, with the sGRCR requiring around 50% of the GRCR’s FLOP count. The sPRIDe also requires around 50% of the PRIDe’s total FLOP count, but the count attained by the PRIDe/sPRIDe is larger than in the case of GRCR/sGRCR.

The numerator and denominator of the FLOP ratios in Table 1 are decomposed into an SCM or PSCM plus test statistic FLOPs, allowing for a clear verification of the large reduction in the FLOP count from the original to simplified test statistics. It can be noticed that the adoption of the PSCM leads to computational costs ranging from 3.37% (for the sAID) to 61.9% (for the sTID) of those incurred by the corresponding SCM-based test statistics.

## 7. Numerical Results

This section presents computer simulation results aimed at comparing the performances of the detectors PRIDe, GID, GRCR, AID, TID, and LMPIT when their test statistics are computed using either the SCM or the PSCM. The simulations were conducted in MATLAB, employing the code provided in [21]. Unless otherwise specified, the default system parameters, which have been selected to reflect practically feasible values in real settings, are summarized in Table 2.

It is important to emphasize that a rigorous theoretical performance analysis of the detectors considered herein requires the statistical characterization of their test statistics. However, the probability density functions of these statistics are not available in the literature, even under the null hypothesis. Their derivation remains a challenging open problem, primarily due to the complexity of the adopted sensing channel model, which departs significantly from the tractable AWGN assumption commonly used in analytical studies. In the absence of such closed-form distributions, the performance evaluation in this work is based on empirical evidence obtained through extensive Monte Carlo simulations.

Although several metrics can be used to evaluate the performance of the spectrum sensing process, here the evaluation is made in terms of the probability of detection, $P_{d}$, as a function of the most relevant system parameters, under the assumption of a constant probability of false alarm. For each detector, the decision threshold is computed independently to ensure that $P_{\mathrm{fa}}=0.1$ is maintained.

**Figure 1:**
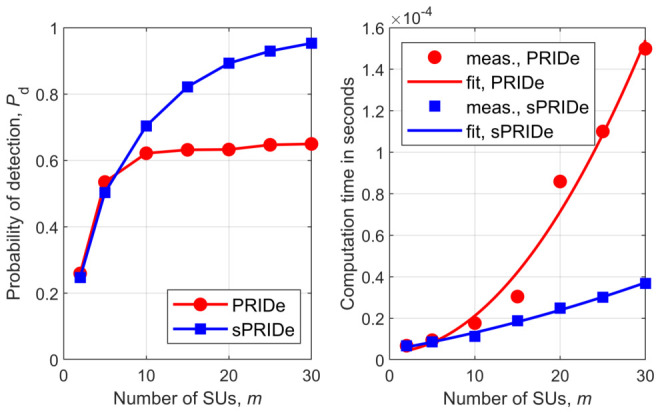
PRIDe and sPRIDe: (**left**) detection probability versus *m*; (**right**) runtime versus *m*.

**Figure 2:**
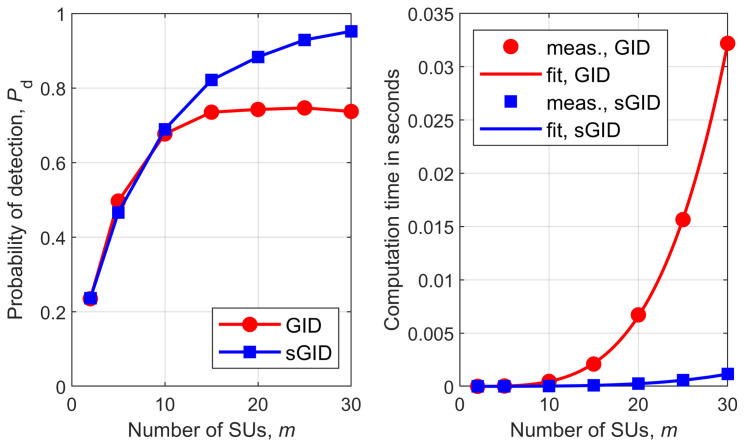
GID and sGID: (**left**) detection probability versus *m*; (**right**) runtime versus *m*.

**Figure 3:**
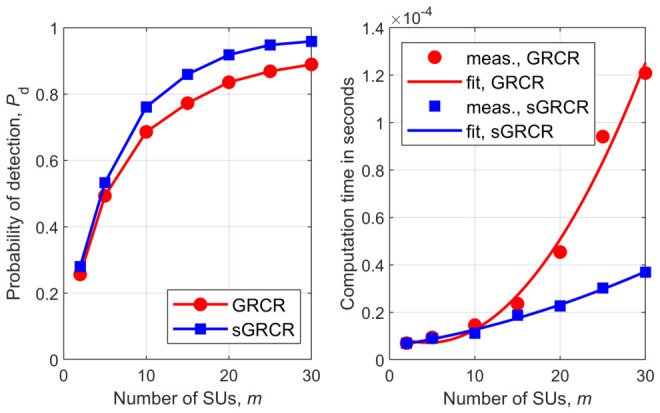
GRCR and sGRCR: (**left**) detection probability versus *m*; (**right**) runtime versus *m*.

**Figure 4:**
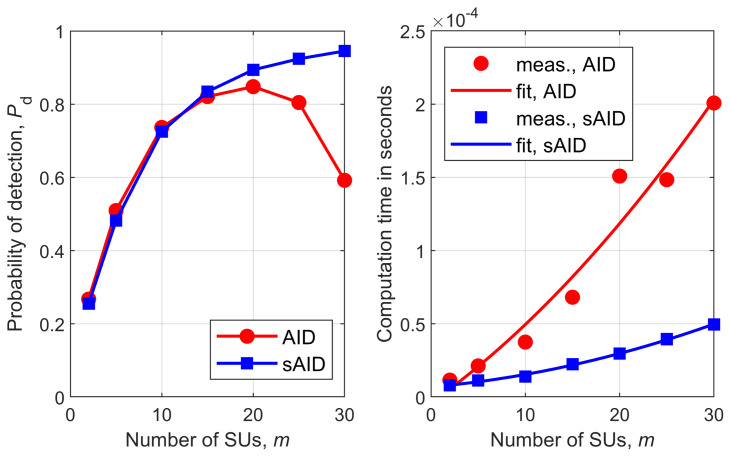
AID and sAID: (**left**) detection probability versus *m*; (**right**) runtime versus *m*.

**Figure 5:**
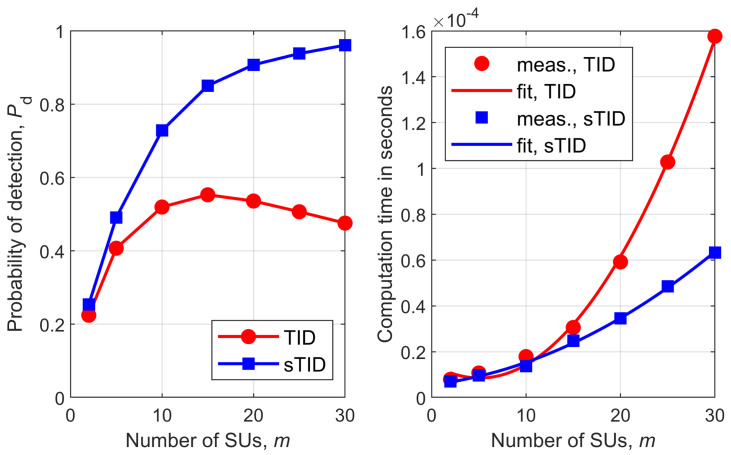
TID and sTID: (**left**) detection probability versus *m*; (**right**) runtime versus *m*.

**Figure 6:**
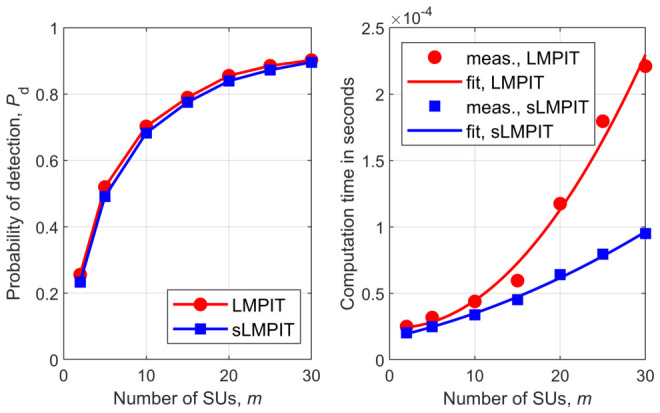
LMPIT and sLMPIT: (**left**) detection probability versus *m*; (**right**) runtime versus *m*.

Figure 1, Figure 2, Figure 3, Figure 4, Figure 5 and Figure 6 show $P_{d}$ as a function of the number of SUs in cooperation, *m*, and the MATLAB runtime, in seconds, versus *m* for all detectors considered in this study. Curve fitting to the measured runtime has been performed for each detector. Measured runtimes and the fitted curves are shown in the right-hand graphs of Figure 1, Figure 2, Figure 3, Figure 4, Figure 5 and Figure 6. The equations for the fitted runtimes are listed in Table 3 for n=100. From this table, it can be seen that the growth rate of the runtimes follow the FLOP count growth rate, as given by the dominant term in each case. The normalized runtimes (with respect to the largest) are also shown in the rightmost column of Table 3 for m=10.

It can be notice from Figure 1, Figure 2, Figure 3, Figure 4, Figure 5 and Figure 6 that all simplified detectors attained superior performance compared to their original versions, except for the LMPIT/sLMPIT (Figure 6), whose performances are approximately equal to one another.

Interestingly enough, Figure 1, Figure 2, Figure 3, Figure 4, Figure 5 and Figure 6 show that, whereas the original detectors attain quite discrepant performances for some values of *m*, the PSCM-based detectors perform close to each other for all *m*. This suggests that the choice of the most attractive detector may be made in light of the runtimes shown in Table 3, which favors sAID, sTID, sPRIDe and sGRCR, with the GRCR closely following. The choice also may be made in light of the computational complexities given in Table 1. For example, when m=10 and n=100 all simplified detectors, except the sGID, require approximately the same number of FLOPs, being equally attractive. A similar analysis must be performed for other values of *m* and *n*.

Another interesting behavior can be observed in Figure 1, Figure 2, Figure 4 and Figure 5 in regard to the non-monotonic increase in $P_{d}$ as *m* increases in the case of SCM-based detectors. Notice that this negative behavior is solved by the PSCM-based detectors. The performance saturation or reduction beyond some value of *m* is credited to the presence of shadowing, meaning that if this impairment is removed, the monotonic increase in $P_{d}$ as *m* increases is restored, except for the AID. As reported in [10], the AID suffers from such non-monotonicity even in the absence of shadowing, depending on the inequality aversion parameter ϵ.

**Figure 7:**
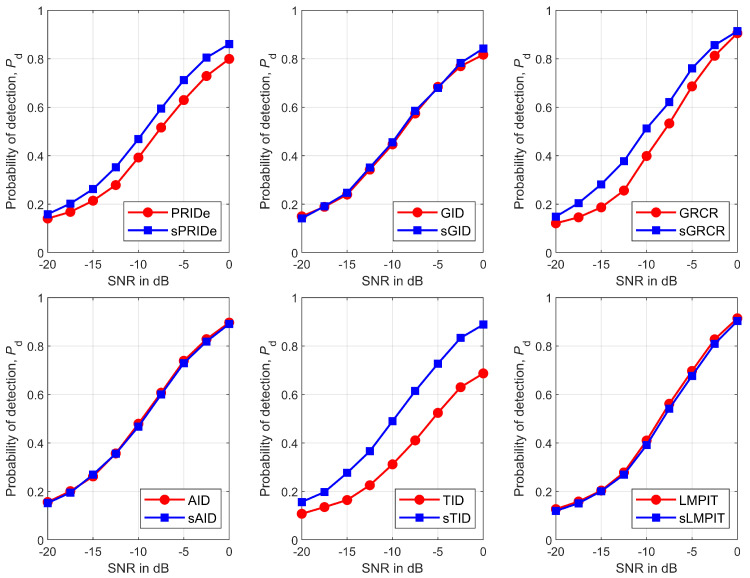
$P_{d}$ versus SNR for PRIDe/sPRIDe (**upper left**), GID/sGID (**upper middle**), GRCR/sGRCR (**upper right**), AID/sAID (**bottom left**), TID/sTID (**bottom middle**), and LMPIT/sLMPIT (**bottom right**).

Figure 7 gives $P_{d}$ versus SNR for all detectors, from where it can be seen that the PSCM-based detectors perform approximately equal to or better than their SCM-based predecessors. As expected, the performance of all detectors monotonically improve as the SNR increases.

**Figure 8:**
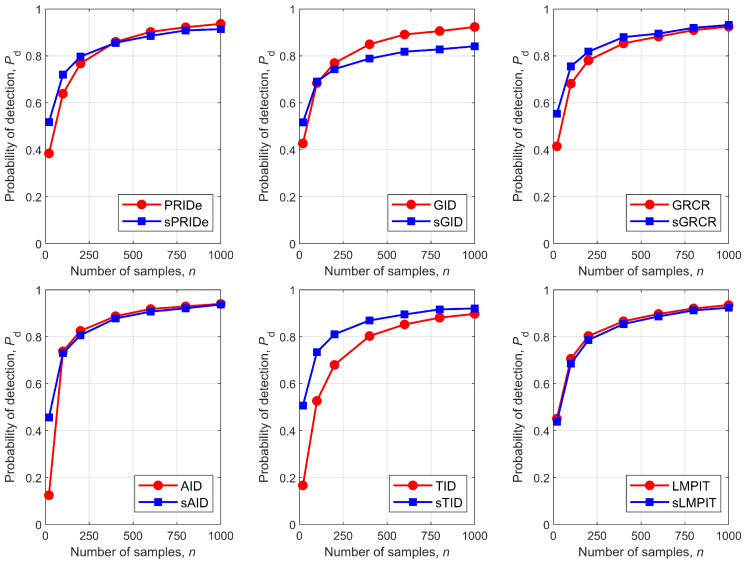
$P_{d}$ versus *n* for PRIDe/sPRIDe (**upper left**), GID/sGID (**upper middle**), GRCR/sGRCR (**upper right**), AID/sAID (**bottom left**), TID/sTID (**bottom middle**), and LMPIT/sLMPIT (**bottom right**).

Figure 8 shows $P_{d}$ as a function of the number of samples, *n*, for all detectors. It can be observed that the relative performance ranking depends on *n*: the PSCM-based detectors generally exhibit superior or comparable performance for smaller values of *n*, whereas the SCM-based detectors tend to outperform or match the PSCM-based detectors as *n* increases. For the sTID, it consistently achieved the best performance across the entire range of *n*. The sGRCR demonstrated a similar trend, with its performance converging to that of the GRCR for large *n*. The LMPIT and sLMPIT yielded approximately the same performance throughout all considered values of *n*. It is worth noting that the PSCM reduces the parameter dimension and thus is more robust in the small-sample regime, while the SCM can exploit weak information in the imaginary part when many samples are available.

**Figure 9:**
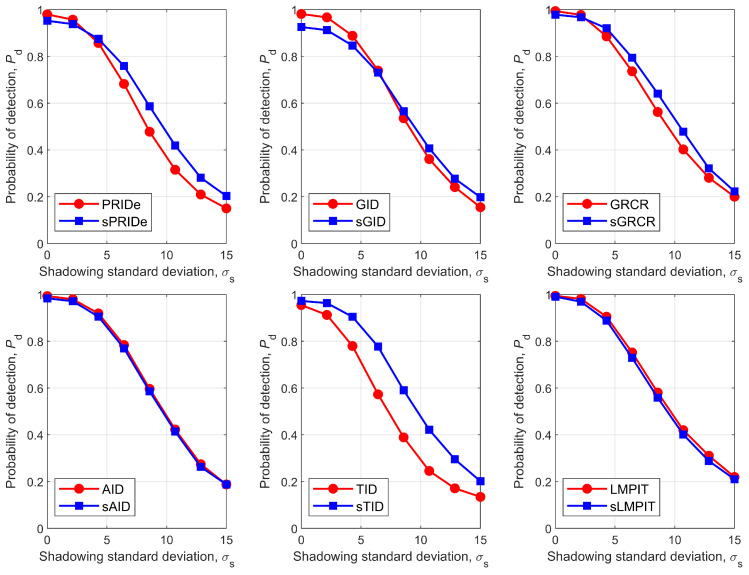
$P_{d}$ versus $\sigma_{s}$ for PRIDe/sPRIDe (**upper left**), GID/sGID (**upper middle**), GRCR/sGRCR (**upper right**), AID/sAID (**bottom left**), TID/sTID (**bottom middle**), and LMPIT/sLMPIT (**bottom right**).

Figure 9 depicts $P_{d}$ as a function of the standard deviation of the shadowing, $\sigma_{s}$. As expected, the performance of all detectors decreases monotonically with increasing $\sigma_{s}$. Once again, the PSCM-based detectors demonstrate superior or equivalent performance compared to the SCM-based counterparts in most scenarios. The SCM-based detectors PRIDe and GID (and to a lesser extent, the GRCR) may surpass the PSCM-based detectors under low values of $\sigma_{s}$.

It is possible to roughly associate the performance degradation caused by shadowing with the corresponding reduction in SNR by comparing Figure 7 and Figure 9. For instance, the transition from no shadowing to shadowing with $\sigma_{s}=7$ dB results in a decrease in $P_{d}$ for the sPRIDe detector from approximately 0.95 to about 0.7, as shown in the upper-left graph of Figure 9, corresponding to a variation of 0.25. By mapping this variation onto the upper-left graph of Figure 7, one finds an SNR difference of approximately −7.5 dB. Therefore, in the absence of shadowing, the sPRIDe detector could achieve $P_{d}=0.85$ with an SNR around −7.5 dB.

**Figure 10:**
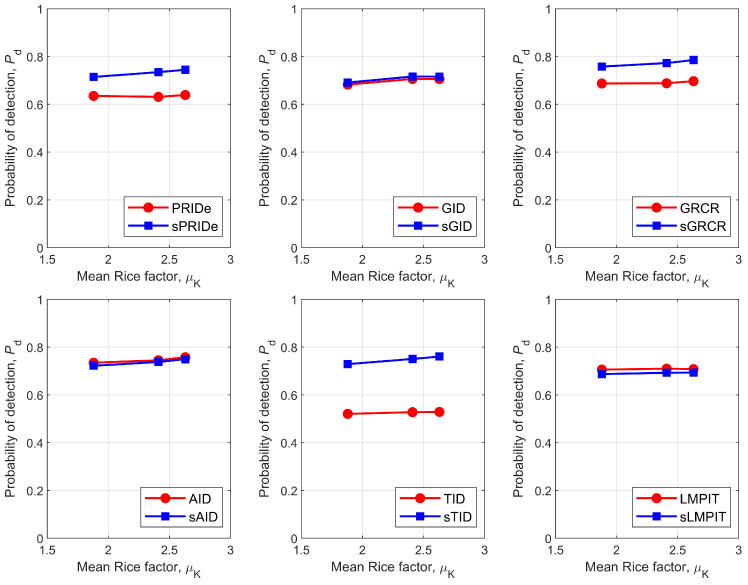
$P_{d}$ versus $\mu_{K}$ for PRIDe/sPRIDe **(upper left**), GID/sGID (**upper middle**), GRCR/sGRCR (**upper right**), AID/sAID (**bottom left**), TID/sTID (**bottom middle**), and LMPIT/sLMPIT (**bottom right**).

The influence of the mean $\mu_{K}$ (and, implicitly, the standard deviation $\sigma_{K}$) of the Rice factor on $P_{d}$ in depicted in Figure 10, considering urban, suburban and rural environments (see Section 2). Taking into account that ≈99.73% of the excursion of the Gaussian random variable $K^{\mathrm{dB}}$ lies in the interval $\left[\mu_{K}-3\sigma_{K},\mu_{K}+3\sigma_{K}\right]$, the overall variation in the Rice factor considered in Figure 10 is approximately from −10.5 dB to 14.3 dB. In practical terms, this means that the line-of-sight condition in the sensing channel dynamically varies from Rayleigh fading to pure AWGN (additive white Gaussian noise).

From Figure 10, it can be observed that the proposed PSCM-based detectors sPRIDe, sGRCR, and sTID outperform their SCM-based counterparts across all evaluated environments. In contrast, the GID/sGID, AID/sAID, and LMPIT/sLMPIT pairs achieve approximately equivalent performance, with the LMPIT exhibiting a slight advantage over the sLMPIT.

### Constant False Alarm Rate

The CFAR is a necessary property of any detector for spectrum sensing. It refers to the ability to maintain the false alarm rate irrespective of the noise variance, meaning that the decision threshold does not change if this variance changes.

All PSCM-based detectors considered herein are constructed from homogeneous functions of the covariance matrix elements, combined with normalization terms that cancel the dependence on noise variance by balancing its contributions in the numerator and denominator. Since the PSCM preserves the same scaling behavior with respect to noise power as the SCM, the resulting test statistics inherit the invariance properties required to ensure the CFAR property.

A simple procedure has been adopted to verify the CFAR property. According to the system model and the simulation setup, the noise variance is determined by the SNR, since the PU transmit power is kept constant. Therefore, by varying the SNR and recording the corresponding decision threshold for each case, the CFAR property is confirmed if the threshold remains invariant with respect to changes in noise variance. This procedure has been applied to all detectors, and the CFAR property has been verified in all cases, as illustrated in Table 4.

The code [21] was used to estimate the decision thresholds for SNR values ranging from −20 dB to 0 dB in steps of 2.5 dB, with 30,000 Monte Carlo realizations per SNR point. The remaining system parameters are listed in Table 2. For each detector, the threshold was obtained as the empirical $\left(1-P_{\mathrm{fa}}\right)$-quantile of the test statistic under the null hypothesis. This approach ensures that the target false alarm probability is satisfied without requiring closed-form expressions for the test statistic distributions, which are not available.

To assess the accuracy of the decision threshold estimates reported in Table 4, let (1−α) denote the confidence level. Since the theoretical distributions of the test statistics under $H_{0}$ are unknown, a distribution-free (1−α)×100% confidence interval for the true threshold can be constructed using order statistics, as described in [25]. For 30,000 Monte Carlo realizations and a confidence level of 95% (α=0.05), the resulting confidence interval exhibits a relative width of approximately 0.1%, indicating a high level of precision in the estimated thresholds.

## 8. Conclusions

This work has proposed simplified test statistics for six covariance-based blind detectors employed in data fusion CSS, introducing the use of a symmetric real-valued partial SCM in place of the full Hermitian SCM. The proposed formulations constitute definitive and practically viable alternatives to the original SCM-based approaches, as they have been shown to significantly reduce computational complexity while approximately maintaining, and in several cases enhancing, detection performance under realistic operating conditions that include non-uniform and time-varying noise powers, distance-dependent path loss, spatially correlated shadowing, and multipath fading characterized by a random Rice factor. The proposed PSCM-based detectors are most suitable under circular or near-circular primary signal conditions, and the SCM-based detectors may be preferable when non-circularity is significant.

A natural direction for future research is the implementation of the proposed detectors in hardware, and the assessment of their performance and complexity in that context. Both the FLOP count and MATLAB runtime presented here offer only partial indications of hardware complexity, as they do not account for data movement, resource contention, or latency in custom implementations. Additionally, MATLAB runtime reflects software-level performance on general-purpose processors and cannot be directly extrapolated to hardware platforms such as field-programmable gate arrays (FPGAs) or application-specific integrated circuits (ASICs).

Another important avenue for future research concerns the development of a comprehensive mathematical framework that rigorously supports the use of the PSCM. In particular, deriving the statistical characterization of the proposed test statistics requires knowledge of their probability density functions under both hypotheses, which in turn demands an extension of random matrix theory to accommodate the structure induced by the PSCM. This task is especially challenging because the underlying sensing channel model departs substantially from the commonly adopted AWGN assumption, incorporating distance-dependent received signal levels, spatially correlated shadowing, non-uniform and time-varying noise powers, and multipath fading with a random Rice factor. These impairments significantly complicate analytical tractability and make closed-form performance analysis considerably more difficult than in conventional settings.

Other additional directions for future research include adapting the proposed PSCM-based detectors for application in non-cooperative spectrum sensing (NCSS), and investigating the performance of both CSS and NCSS in the presence of typical hardware impairments inherent to a direct-conversion receiver (DCR) architecture when employed at the SUs.

## Figures and Tables

**Table 1:** Approximate numbers of FLOPs.

Detector	Number of FLOPs of (R or S) + (Test Statistic)	FLOP Ratio (*m* = 10, *n* = 100)
PRIDe	$\left(4m^{2}n-m^{2}+4mn-m\right)+\left(12m^{2}-1\right)$	$\frac{21945+400}{43890+1199}=\frac{22345}{45089}=0.4956$
sPRIDe	$\left(2m^{2}n+2mn-m^{2}/2-m/2\right)+\left(4m^{2}\right)$	
GID	$\left(4m^{2}n-m^{2}+4mn-m\right)+\left(7m^{4}+5m^{2}\right)$	$\frac{21945+15249}{43890+70500}=\frac{37194}{114390}=0.3252$
sGID	$\left(2m^{2}n+2mn-m^{2}/2-m/2\right)+\left(3m^{4}/2+5m^{2}/2-1\right)$	
GRCR	$\left(4m^{2}n-m^{2}+4mn-m\right)+\left(5m^{2}+m\right)$	$\frac{21945+54}{43890+510}=\frac{21999}{44400}=0.4955$
sGRCR	$\left(2m^{2}n+2mn-m^{2}/2-m/2\right)+\left(m^{2}/2+m/2-1\right)$	
AID	$\left(4m^{2}n-m^{2}+4mn-m\right)+\left(40m^{2}+7\right)$	$\frac{21945+135}{43890+4007}=\frac{22080}{47897}=0.4610$
sAID	$\left(2m^{2}n+2mn-m^{2}/2-m/2\right)+\left(3m^{2}/2-3m/2\right)$	
TID	$\left(4m^{2}n-m^{2}+4mn-m\right)+\left(13m^{2}/2+9m/2-1\right)$	$\frac{21945+430}{43890+694}=\frac{22375}{44584}=0.5019$
sTID	$\left(2m^{2}n+2mn-m^{2}/2-m/2\right)+\left(4m^{2}+3m\right)$	
LMPIT	$\left(4m^{2}n-m^{2}+4mn-m\right)+\left(8m^{2}+2m-1\right)$	$\frac{21945+419}{43890+819}=\frac{22445}{44789}=0.5012$
sLMPIT	$\left(2m^{2}n+2mn-m^{2}/2-m/2\right)+\left(4m^{2}+2m-1\right)$	

**Table 2:** Default system parameters.

Parameter	Symbol	Value
Number of SUs	*m*	10
Average signal-to-noise ratio	SNR	−5 dB
Number of Monte Carlo runs	—	10,000
Path loss exponent	η	2.5
Normalized coverage radius	*r*	1 m
Reference distance in path loss model	$d_{0}$	0.001 m
PU transmit power	$P_{\mathrm{tx}}$	5 W
PU transmitter location	(x,y)	(r,r) m
Number of samples per SU	*n*	100
Samples per QPSK symbol	$N_{s}$	n/10
Noise level variation fraction	ρ	0.5
Mean of Rice factor	$\mu_{K}$	1.88 dB
Std. deviation of Rice factor	$\sigma_{K}$	4.13 dB
Reference false alarm probability	$P_{\mathrm{fa}}$	0.1
Inequality aversion parameter of AID	ϵ	0.01
Shadowing standard deviation	$\sigma_{s}$	7 dB
Size of Σ, $S_{u}$, $S_{c}$	—	50×50
Spatial correlation length	Λ	40

**Table 3:** Fitted equations for n=100, and normalized runtime for m=10.

Detector	Fitted Equation for Total Runtime, in Seconds	Normalized Runtime
PRIDe	$t\left(m\right)=9.0\times 10^{-8}m^{2}+6.6\times 10^{-7}m+4.5\times 10^{-6}$	0.0563
sPRIDe	$t\left(m\right)=1.7\times 10^{-8}m^{2}+4.7\times 10^{-7}m+6.4\times 10^{-6}$	0.0359
GID	$t\left(m\right)=3.6\times 10^{-8}m^{4}+3.7\times 10^{-6}m^{2}-3.8\times 10^{-5}m+6.9\times 10^{-6}$	1.0000
sGID	$t\left(m\right)=1.3\times 10^{-9}m^{4}+1.8\times 10^{-7}m^{2}-6.1\times 10^{-7}m+6.9\times 10^{-6}$	0.0891
GRCR	$t\left(m\right)=7.1\times 10^{-8}m^{2}+3.2\times 10^{-7}m+6.1\times 10^{-6}$	0.0460
sGRCR	$t\left(m\right)=1.1\times 10^{-8}m^{2}+5.8\times 10^{-7}m+6.1\times 10^{-6}$	0.0359
AID	$t\left(m\right)=7.5\times 10^{-8}m^{2}+4.3\times 10^{-6}m-1.7\times 10^{-6}$	0.1234
sAID	$t\left(m\right)=2.2\times 10^{-8}m^{2}+7.5\times 10^{-7}m-6.6\times 10^{-6}$	0.0280
TID	$t\left(m\right)=1.7\times 10^{-7}m^{2}-1.1\times 10^{-6}m+1.1\times 10^{-5}$	0.0728
sTID	$t\left(m\right)=4.5\times 10^{-8}m^{2}+4.6\times 10^{-7}m+6.2\times 10^{-6}$	0.0336
LMPIT	$t\left(m\right)=1.9\times 10^{-7}m^{2}+3.6\times 10^{-7}m+2.5\times 10^{-5}$	0.1344
sLMPIT	$t\left(m\right)=3.3\times 10^{-8}m^{2}+1.6\times 10^{-6}m+1.7\times 10^{-5}$	0.1011

**Table 4:** Verification of the CFAR property of PRIDe and sPRIDe.

Quantity	Values
Average noise variances	$4.5\times 10^{-5}$, $2.5\times 10^{-5}$, $1.4\times 10^{-5}$, $8.0\times 10^{-6}$, $4.5\times 10^{-6}$, $2.5\times 10^{-6}$, $1.4\times 10^{-6}$, $8.0\times 10^{-7}$, $4.5\times 10^{-7}$
PRIDe thresholds using R	$8.93\times 10^{-1}$, $8.93\times 10^{-1}$, $8.93\times 10^{-1}$, $8.93\times 10^{-1}$, $8.93\times 10^{-1}$, $8.93\times 10^{-1}$, $8.93\times 10^{-1}$, $8.93\times 10^{-1}$, $8.93\times 10^{-1}$
sPRIDe thresholds using S	$6.16\times 10^{-1}$, $6.16\times 10^{-1}$, $6.15\times 10^{-1}$, $6.16\times 10^{-1}$, $6.15\times 10^{-1}$, $6.16\times 10^{-1}$, $6.16\times 10^{-1}$, $6.16\times 10^{-1}$, $6.16\times 10^{-1}$
GID thresholds using R	$1.15\times 10^{0}$, $1.15\times 10^{0}$, $1.15\times 10^{0}$, $1.15\times 10^{0}$, $1.15\times 10^{0}$, $1.15\times 10^{0}$, $1.15\times 10^{0}$, $1.15\times 10^{0}$, $1.15\times 10^{0}$
sGID thresholds using S	$9.31\times 10^{-3}$, $9.31\times 10^{-3}$, $9.32\times 10^{-3}$, $9.31\times 10^{-3}$, $9.32\times 10^{-3}$, $9.32\times 10^{-3}$, $9.32\times 10^{-3}$, $9.31\times 10^{-3}$, $9.32\times 10^{-3}$
GRCR thresholds using R	$8.59\times 10^{-1}$, $8.59\times 10^{-1}$, $8.58\times 10^{-1}$, $8.59\times 10^{-1}$, $8.58\times 10^{-1}$, $8.60\times 10^{-1}$, $8.58\times 10^{-1}$, $8.59\times 10^{-1}$, $8.60\times 10^{-1}$
sGRCR thresholds using S	$6.10\times 10^{-2}$, $6.10\times 10^{-2}$, $6.11\times 10^{-2}$, $6.11\times 10^{-2}$, $6.20\times 10^{-2}$, $6.20\times 10^{-2}$, $6.20\times 10^{-2}$, $6.20\times 10^{-2}$
AID thresholds using R	$1.03\times 10^{2}$, $1.03\times 10^{2}$, $1.03\times 10^{2}$, $1.03\times 10^{2}$, $1.03\times 10^{2}$, $1.03\times 10^{2}$, $1.03\times 10^{2}$, $1.03\times 10^{2}$, $1.03\times 10^{2}$
sAID thresholds using S	$3.61\times 10^{1}$, $3.61\times 10^{1}$, $3.60\times 10^{1}$, $3.61\times 10^{1}$, $3.60\times 10^{1}$, $3.61\times 10^{1}$, $3.60\times 10^{1}$, $3.61\times 10^{1}$, $3.61\times 10^{1}$
TID thresholds using R	$1.46\times 10^{-2}$, $1.46\times 10^{-2}$, $1.46\times 10^{-2}$, $1.46\times 10^{-2}$, $1.46\times 10^{-2}$, $1.46\times 10^{-2}$, $1.47\times 10^{-2}$, $1.47\times 10^{-2}$, $1.47\times 10^{-2}$
sTID thresholds using S	$5.06\times 10^{-3}$, $5.06\times 10^{-3}$, $5.07\times 10^{-3}$, $5.07\times 10^{-3}$, $5.06\times 10^{-3}$, $5.07\times 10^{-3}$, $5.07\times 10^{-3}$, $5.07\times 10^{-3}$, $5.07\times 10^{-3}$
LMPIT thresholds using R	$1.11\times 10^{1}$, $1.11\times 10^{1}$, $1.11\times 10^{1}$, $1.11\times 10^{1}$, $1.11\times 10^{1}$, $1.11\times 10^{1}$, $1.11\times 10^{1}$, $1.11\times 10^{1}$, $1.11\times 10^{1}$
sLMPIT thresholds using S	$1.06\times 10^{1}$, $1.06\times 10^{1}$, $1.06\times 10^{1}$, $1.06\times 10^{1}$, $1.06\times 10^{1}$, $1.06\times 10^{1}$, $1.06\times 10^{1}$, $1.06\times 10^{1}$, $1.06\times 10^{1}$

## Data Availability

The original contributions presented in this study are included in the article. Further inquiries can be directed to the author.

## Abbreviations

The following abbreviations are used in this manuscript:

AGM	arithmetic to geometric mean
AID	Atkinson index detector
ASIC	application-specific integrated circuit
AWGN	additive white Gaussian noise
CAV	covariance absolute value
CFAR	constant false alarm rate
CFD	cyclostationary feature detector
CR	cognitive radio
CSS	cooperative spectrum sensing
DCR	decision combining rule
ED	energy detector
FC	fusion center
FPGA	field-programmable gate array
GID	Gini index detector
GRCR	Gerschgorin radii and centers ratio
HR	Hadamard ratio
LMPIT	locally most powerful invariant test
MED	maximum eigenvalue detector
MF	matched filter
MGF	moment generating function
MMED	maximum–minimum eigenvalue detector
MSEE	mean-to-square extreme eigenvalue
NCSS	non-cooperative spectrum sensing
PRIDe	Pietra-Ricci index detector
PSCM	partial sample covariance matrix
PU	primary user
QPSK	quaternary phase-shift keying
RF	radio frequency
SCM	sample covariance matrix
SLE	scaled largest eigenvalue
SNR	signal-to-noise ratio
SU	secondary user
TID	Theil index detector

## Appendix A. Expected Value of *P*(*d*)

The distance-dependent area-mean signal power received by a node at a distance *d* from the transmitter in a wireless communication system can be calculated as

$$P\left(d\right)=P_{\mathrm{tx}}{\left(\frac{d}{d_{0}}\right)}^{-\eta },$$

where $P_{\mathrm{tx}}$ is the transmit power, $d_{0}$ is the reference distance above the near-field region of the transmit antenna, and η is the path loss exponent.

Assuming that the coordinates of a node are (x,y), and that the transmitter is at $\left(x_{\mathrm{tx}},y_{\mathrm{tx}}\right)$, the distance *d* is given by

$$d=\sqrt{{\left(x-x_{\mathrm{tx}}\right)}^{2}+{\left(y-y_{\mathrm{tx}}\right)}^{2}}.$$

Since the nodes are uniformly distributed within a circular area of radius *r*, any point within the circle can be represented in polar coordinates as (z,θ), for z∈[0,r] and θ∈[0,2π), where x=zcosθ and y=zsinθ. Hence, the distance *d* can be written as

$$d=\sqrt{{\left(z\cos\theta -x_{\mathrm{tx}}\right)}^{2}+{\left(z\sin\theta -y_{\mathrm{tx}}\right)}^{2}}.$$

The expected value E[P(d)] is obtained by integrating P(d) over the circular area and normalizing the result by the area of the circle; that is,

$$E\left[P\left(d\right)\right]=\frac{1}{\pi r^{2}}\int_{0}^{2\pi }\int_{0}^{r}P_{\mathrm{tx}}{\left(\frac{d}{d_{0}}\right)}^{-\eta }z\;dz\;d\theta ,$$

where zdzdθ is the infinitesimal area element in polar coordinates. Substituting the expression for *d*, it follows that

$$\begin{array}{cc} E[P(d)] & =\frac{P_{\mathrm{tx}}}{\pi r^{2}d_{0}^{-\eta }}\int_{0}^{2\pi }\int_{0}^{r}\left[{\left(z\cos\theta -x_{\mathrm{PU}}\right)}^{2}\right.\\ \; & \;{\left.+{\left(z\sin\theta -y_{\mathrm{PU}}\right)}^{2}\right]}^{-\frac{\eta }{2}}z\;dz\;d\theta . \end{array}$$

The double integral in the above expression, though in closed form, is complex to solve analytically and may require numerical methods.

## Appendix B. Expected Value of 10^*S*/10^

Let *S* be a Gaussian random variable with zero mean and variance $\sigma_{s}^{2}$. The moment-generating function (MGF) of *S* is

$$M_{S}\left(t\right)=E\left[e^{tS}\right]=\exp\left(\frac{\sigma_{s}^{2}t^{2}}{2}\right).$$

Recognizing that $10^{S/10}=e^{S\ln(10)/10}$, $E\left[10^{S/10}\right]=E\left[e^{S\ln(10)/10}\right]$. This is equivalent to evaluating the MGF of *S* at t=ln(10)/10; that is

$$E\left[10^{S/10}\right]=M_{S}\left(\frac{\ln(10)}{10}\right)=\exp\left[\frac{\sigma_{s}^{2}}{2}{\left(\frac{\ln(10)}{10}\right)}^{2}\right],$$

which finally yields

$$E\left[10^{S/10}\right]=\exp\left(\frac{\sigma_{s}^{2}\ln^{2}\left(10\right)}{200}\right).$$

## Appendix C. Expected Value of 1/*σ*^2^

The expected value of $1/\sigma^{2}=1/\left[\left(1+\rho U\right){\bar{\sigma }}^{2}\right]$ is the expected value of a function of the uniform random variable U∼U[−1,1], which is

$$E\left[\frac{1}{\sigma^{2}}\right]=\frac{1}{2{\bar{\sigma }}^{2}}\int_{-1}^{1}\frac{1}{1+\rho u}\;du.$$

Performing the substitution t=1+ρu, dt=ρdu and du=dt/ρ. When u=−1, t=1−ρ, and when u=1, t=1+ρ. Thus, it follows that

$$E\left[\frac{1}{\sigma^{2}}\right]=\frac{1}{2{\bar{\sigma }}^{2}\rho }\int_{1-\rho }^{1+\rho }\frac{1}{t}dt.$$

Since the integral of 1/t is ln|t|, after some simple manipulations, it is found that

$$E\left[\frac{1}{\sigma^{2}}\right]=\frac{1}{2{\bar{\sigma }}^{2}\rho }\ln\left(\frac{1+\rho }{1-\rho }\right)$$

and

$$E\left[\frac{1}{\sigma^{2}}\right]=\frac{1}{{\bar{\sigma }}^{2}}$$

for 0<ρ<1 and ρ=0, respectively.

## Appendix D. FLOP Count Analysis

This appendix presents detailed (though approximate) computational complexity analysis of the test statistics considered in this study. The complexity is measured by the total number of FLOPs required to compute each test statistic from the received signal samples in Y, based on standard arithmetic cost models. Each basic real operation (e.g., addition, multiplication, and division) is counted as one FLOP, while compound operations such as complex multiplication or modulus are decomposed into their constituent real operations using established FLOP-equivalent estimates.

It must be highlighted that the FLOP count estimates are not accurate on general-purpose processors and vary significantly across hardware platforms. Hence, the FLOP counts are rough approximations, as the actual counts depend on the specific implementation. A more conservative estimate could be provided, but this modification would not alter the relative complexity ordering.

### Appendix D.1. FLOP Count to Compute ***R***, Not Exploring the Hermitian Property

Each entry of $R=YY^{†}$ is $R_{ij}=\sum_{k=1}^{n}Y_{ik}Y_{jk}^{*}$. One complex multiplication requires 6 real FLOPs (4 for multiplications and 2 for additions), and one complex addition requires 2 real FLOPs. To compute the inner product *n* complex multiplications are required, yielding 6n FLOPs, and n−1 complex additions, yielding 2(n−1) FLOPs. Hence, each $R_{ij}$ needs 6n+2(n−1)=8n−2 FLOPs. The total cost to compute the $m^{2}$ elements of R is $\left(8n-2\right)m^{2}=8m^{2}n-2m^{2}$.

### Appendix D.2. FLOP Count to Compute ***R***, Exploring the Hermitian Property

Each entry of $R=YY^{†}$ is given by $R_{ij}=\sum_{k=1}^{n}Y_{ik}Y_{jk}^{*}$. One complex multiplication requires 6 real FLOPs (4 for real multiplications and 2 for additions), and one complex addition requires 2 real FLOPs. To compute the inner product, we need *n* complex multiplications (yielding 6n FLOPs) and n−1 complex additions (yielding 2(n−1) FLOPs). Therefore, computing each entry $R_{ij}$ requires 6n+2(n−1)=8n−2 real FLOPs. Since R is Hermitian, we only need to compute the upper (or lower) triangular part, including the diagonal. The remaining entries are obtained by conjugate symmetry, without any additional FLOPs. The number of unique entries in the upper triangle is m(m+1)/2. Thus, the total cost to compute R by taking advantage of its Hermitian structure is $\left(8n-2\right)m(m+1)/2=4m^{2}n-m^{2}+4mn-m$

### Appendix D.3. FLOP Count to Compute ***S***, Not Exploring the Symmetry Property

To compute $S=ℜ\left(Y\right)ℜ{\left(Y\right)}^{T}+ℑ\left(Y\right)ℑ{\left(Y\right)}^{T}$ one needs two real matrix multiplications and a matrix addition. The computation of a dot product of two real vectors of length *n* costs *n* multiplications plus n−1 additions, yielding 2n−1 FLOPs. For the $m^{2}$ elements (not exploring matrix symmetry), the cost is $\left(2n-1\right)m^{2}$ for each matrix multiplication. The addition of the matrices costs $m^{2}$ FLOPs. Hence, the total cost to compute S is $2m^{2}\left(2n-1\right)+m^{2}=4m^{2}n-m^{2}$, which is half of the cost to compute R. This is consistent with the fact that R=S+jT, where T has the same computational complexity as S. However, S is significantly simpler for hardware implementation due to real-only operations.

### Appendix D.4. FLOP Count to Compute ***S***, Exploring the Symmetry Property

As above, to compute $S=ℜ\left(Y\right)ℜ{\left(Y\right)}^{⊤}+ℑ\left(Y\right)ℑ{\left(Y\right)}^{⊤}$, one needs two real matrix multiplications and a matrix addition. The dot product of two real vectors of length *n* costs *n* multiplications plus n−1 additions, totaling 2n−1 FLOPs. Since S is symmetric, only the upper (or lower) triangular part, including the diagonal, needs to be computed, and the rest is filled by symmetry. The number of unique elements in the upper triangle is m(m+1)/2. For each of the two matrix products, the total cost is (2n−1)m(m+1)/2. The addition of the two partial results involves the same number of entries and costs m(m+1)/2 FLOPs. Hence, the total cost to compute S is $2\left(2n-1\right)m\left(m+1\right)/2+m\left(m+1\right)/2=2m^{2}n+2mn-m^{2}/2-m/2$. This expression shows the computational saving achieved by exploiting the symmetry of S, reducing the cost from $4m^{2}n-m^{2}$ FLOPs (when symmetry is not used) to $2m^{2}n+2mn-m^{2}/2-m/2$ FLOPs. This highlights the benefit of leveraging matrix structure along with real-valued operations.

### Appendix D.5. FLOP Count of PRIDe

- Computation of R: $8m^{2}n-2m^{2}$ FLOPs.
- Mean $\bar{r}=\frac{1}{m^{2}}\sum r_{i}$: One sum with $m^{2}-1$ complex additions, with each addition costing 2 FLOPs. One scalar division by $m^{2}$ requires 1 FLOP. The cost is $m^{2}-1+1=m^{2}$.
- First sum $\sum |r_{i}|$: Complex absolute value $\left|a+jb\right|=\sqrt{a^{2}+b^{2}}$ requires approximately 4 FLOPs per entry (2 multiplications, 1 addition, and 1 square root). The summation needs $\left(m^{2}-1\right)$ additions. The cost is $4m^{2}+\left(m^{2}-1\right)=5m^{2}-1$.
- Second sum $\sum |r_{i}-\bar{r}|$: 1 FLOP for real subtraction plus 4 FLOPs per magnitude, yielding 5 FLOPs per element across $m^{2}$ elements, plus $m^{2}-1$ additions, yields $6m^{2}-1$ FLOPs.
- Final division costing 1 FLOP.
- Total computational complexity of PRIDe: $8m^{2}n-2m^{2}+12m^{2}-1=8m^{2}n+10m^{2}-1$ FLOPs.

### Appendix D.6. FLOP Count of sPRIDe

- Computation of S: $4m^{2}n-m^{2}$ FLOPs.
- Mean $\bar{s}=\frac{1}{m^{2}}\sum s_{i}$: $m^{2}-1$ additions, plus 1 scalar division, yielding $m^{2}$ FLOPs.
- Numerator $\sum s_{i}$: Reuse from above, meaning no additional cost.
- Denominator $\sum |s_{i}-\bar{s}|$: $m^{2}$ FLOPs for subtractions, $m^{2}$ FLOPs for absolute values, and $m^{2}-1$ FLOPs for additions, yielding $3m^{2}-1$ FLOPs.
- Final division: 1 FLOP.
- Total computational complexity of sPRIDe: $4m^{2}n-m^{2}+4m^{2}=4m^{2}n+3m^{2}$ FLOPs.

### Appendix D.7. FLOP Count of GID

- Computation of R: $8m^{2}n-2m^{2}$ FLOPs.
- Numerator $2\left(m^{2}-m\right)\sum \left|r_{i}\right|$: Complex absolute value $\left|a+jb\right|=\sqrt{a^{2}+b^{2}}$ requires approximately 4 FLOPs per entry. The summation needs $\left(m^{2}-1\right)$ additions, and the multiplication by $2(m^{2}-m)$ requires 1 FLOP. The cost is $4m^{2}+\left(m^{2}-1\right)+1=5m^{2}$ FLOPs.
- Denominator $\sum_{u=1}^{m^{2}}\sum_{j=1}^{m^{2}}\left|r_{u}-r_{j}\right|$: Each subtraction between complex scalars costs 2 FLOPs (real and imaginary parts), and each absolute value costs approximately 4 FLOPs, yielding 6 FLOPs per pairwise difference. There are $m^{4}$ such comparisons plus the cost of $m^{4}-1$ additions, yielding $6m^{4}+m^{4}-1=7m^{4}-1$ FLOPs.
- Final division: 1 FLOP.
- Total computational complexity of GID: $8m^{2}n-2m^{2}+5m^{2}+7m^{4}-1+1=8m^{2}n+7m^{4}+3m^{2}$ FLOPs.

### Appendix D.8. FLOP Count of sGID

- Computation of S: $4m^{2}n-m^{2}$ FLOPs.
- Numerator $\sum s_{i}$: A simple summation over $m^{2}$ real numbers requires $\left(m^{2}-1\right)$ additions.
- Triangular double summation $\sum_{u=1}^{m^{2}}\sum_{j=u}^{m^{2}}\left|s_{u}-s_{j}\right|$: Each pairwise difference costs 1 subtraction and 1 absolute value, yielding 2 FLOPs per pair. The number of such operations is $(m^{2}\left(m^{2}+1\right))/2$. Therefore, the cost is twice this value, that is, $m^{4}+m^{2}$. There are $(m^{2}\left(m^{2}+1\right))/2-1$ additions. Hence, the cost is $m^{4}+m^{2}+\left(m^{2}\left(m^{2}+1\right)\right)/2-1=3m^{4}/2+3m^{2}/2-1$.
- Final division: 1 FLOP.
- Total computational complexity of sGID: $4m^{2}n-m^{2}+m^{2}-1+3m^{4}/2+3m^{2}/2-1+1=4m^{2}n+3m^{4}/2+3m^{2}/2-1$ FLOPs.

### Appendix D.9. FLOP Count of GRCR

- Computation of R: $8m^{2}n-2m^{2}$ FLOPs.
- Numerator: For the inner sum, for each *i* compute $\sum_{j=1}^{m}\left|R_{ij}\right|$. Each $|R_{ij}|$ requires 4 FLOPs and *m* elements, yielding 4m FLOPs per row. Addition of *m* values requires m−1 FLOPs. Subtraction of $R_{ii}$ requires 1 FLOP. Hence, the cost per row is 4m+(m−1)+1=5m. For all rows, the cost is $m5m=5m^{2}$.
- Denominator sum $\sum_{i=1}^{m}R_{ii}$: m−1 additions.
- Final division: 1 FLOP.
- Total computational complexity of GRCR: $8m^{2}n-2m^{2}+5m^{2}+m-1+1=8m^{2}n+3m^{2}+m$ FLOPs.

### Appendix D.10. FLOP Count of sGRCR

- Computation of S: $4m^{2}n-m^{2}$ FLOPs.
- Numerator $\sum_{i=1}^{m}\sum_{j=i+1}^{m}S_{ij}$: There are m(m+1)/2 elements above the diagonal, yielding m(m+1)/2−1 sums.
- Denominator $\sum_{i=1}^{m}S_{ii}$: A summation over *m* elements requires m−1 additions.
- Final division: 1 FLOP.
- Total computational complexity of sGRCR: $4m^{2}n-m^{2}+m\left(m+1\right)/2-1+m-1+1=4m^{2}n-m^{2}/2+m/2-1$ FLOPs.

### Appendix D.11. FLOP Count of AID

- Computation of R: $8m^{2}n-2m^{2}$ FLOPs.
- Numerator: A complex power $x^{\alpha }$ (non-integer real exponent) is computed as $x^{\alpha }=e^{\alpha \log x}$, which involves 1 complex logarithm costing approximately 15 FLOPs, 1 scalar multiplication costing approximately 6 FLOPs, and 1 complex exponential costing approximately 15 FLOPs, yielding 36 FLOPs for each complex power in the numerator, plus $m^{2}-1$ additions and plus 10 FLOPs for the final real exponentiation, yielding $36m^{2}+\left(2m^{2}-2\right)+10=38m^{2}+8$ FLOPs. Note that $\sum_{1,j}R_{ij}^{\alpha }$ is real because $R_{ij}$ and $R_{ji}$ satisfy $R_{ij}^{\alpha }+R_{ji}^{\alpha }=2{\left|R_{ij}\right|}^{\alpha }\cos\left(\alpha \theta_{ij}\right)\in R$, where $\theta_{ij}$ is the argument of $R_{ij}$ and α=1−ϵ.
- Denominator sum $\sum_{i=1}^{m}\sum_{j=1}^{m}R_{ij}$: Summation over $m^{2}$ complex values. Each addition costs 2 FLOPs. Total: $2m^{2}-2$ FLOPs.
- Final scalar division: 1 FLOP.
- Total computational complexity of AID: $8m^{2}n-2m^{2}+38m^{2}+8+2m^{2}-2+1=8m^{2}n+38m^{2}+7$ FLOPs.

### Appendix D.12. FLOP Count of sAID

- Computation of S: $4m^{2}n-m^{2}$ FLOPs.
- Numerator: Assuming J=m(m+1)/2, it follows that there are *J* square roots, J−1 additions and a real multiplication (squaring). Then, the cost of the numerator is J+(J−1)+1=2J=m(m+1).
- Denominator: J−1 additions, yielding m(m+1)/2−1 FLOPs.
- Final scalar division: 1 FLOP.
- Total computational complexity of sAID: $4m^{2}n-m^{2}+2J+\left(J-1\right)+1=3J=4m^{2}n+m^{2}/2-3m/2$ FLOPs.

### Appendix D.13. FLOP Count of TID

- Computation of R: $8m^{2}n-2m^{2}$ FLOPs.
- Mean μ: Summing $m^{2}$ complex values requires $2(m^{2}-1)$ FLOPs, plus one scalar division. Total: $2m^{2}-1$ FLOPs.
- Denominator: The sum has m(m+1)/2 terms. $|R_{ij}|$ requires 4 FLOPs. Division by μ requires 1 FLOP. Logarithm also requires 1 FLOP. Multiplication between $|R_{ij}|$ and the logarithm requires 1 FLOP, and multiplication by $\left(2-\delta_{ij}\right)$ needs 1 FLOP. Hence, 8 FLOPs are required per term. The sum over m(m+1)/2 terms requires m(m+1)/2−1 FLOPs. In total, $8\left[m\left(m+1\right)/2\right]+m\left(m+1\right)/2-1=9m^{2}/2+9m/2-1$ FLOPs are required.
- Final scalar division: 1 FLOP.
- Total computational complexity of TID: $8m^{2}n-2m^{2}+2m^{2}-1+9m^{2}/2+9m/2-1+1=8m^{2}n+9m^{2}/2+9m/2-1$ FLOPs.

### Appendix D.14. FLOP Count of sTID

- Computation of S: $4m^{2}n-m^{2}$ FLOPs.
- Mean $\bar{s}$: Summing $m^{2}$ real elements ($m^{2}-1$ additions) and dividing by the scalar (1 FLOP). Total: $m^{2}$ FLOPs.
- Denominator: m(m+1)/2 terms. $|S_{ij}|$ requires 1 FLOP. Division by $\bar{s}$ requires 1 FLOP. Logarithm requires 1 FLOP. Multiplication between $S_{ij}$ and the logarithm requires 1 FLOP, and multiplication by $\left(2-\delta_{ij}\right)$ needs 1 FLOP. Hence, 5 FLOPs are required per term. The sum over m(m+1)/2 terms requires m(m+1)/2−1 FLOPs. In total, $5\left[m\left(m+1\right)/2\right]+m\left(m+1\right)/2-1=3m^{2}+3m-1$ FLOPs are required.
- Final scalar division: 1 FLOP.
- Total computational complexity of sTID: $4m^{2}n-m^{2}+m^{2}+3m^{2}+3m-1+1=4m^{2}n+3m^{2}+3m$ FLOPs.

### Appendix D.15. FLOP Count of LMPIT

- Computation of R: $8m^{2}n-2m^{2}$ FLOPs.
- Inverse square root of diagonal elements: For each of the *m* entries, compute the square root and reciprocal (2 FLOPs). Total: 2m.
- Matrix normalization C: Each $C_{ij}=R_{ij}/\sqrt{R_{ii}R_{jj}}$ costs ∼4 FLOPs. For $m^{2}$ elements: $4m^{2}$.
- Final sum: For each complex $C_{ij}$, $|C_{ij}{|}^{2}$ costs 3 FLOPs. Summing all $m^{2}$ values costs $m^{2}-1$ additions. Total: $4m^{2}-1$.
- Total computational complexity of LMPIT: $8m^{2}n-2m^{2}+2m+4m^{2}+4m^{2}-1=8m^{2}n+6m^{2}+2m-1$ FLOPs.

### Appendix D.16. FLOP Count of sLMPIT

- Computation of S: $4m^{2}n-m^{2}$ FLOPs.
- Inverse square roots: For *m* entries, each requires 2 FLOPs (sqrt + reciprocal). Total: 2m.
- Matrix normalization C: Each $C_{ij}=S_{ij}/\sqrt{S_{ii}S_{jj}}$ costs ∼2 FLOPs. For $m^{2}$ elements: $2m^{2}$.
- Final sum: For each real $C_{ij}$, compute $C_{ij}^{2}$ (1 FLOP), plus $m^{2}-1$ additions. Total: $2m^{2}-1$.
- Total computational complexity of sLMPIT: $4m^{2}n-m^{2}+2m+2m^{2}+2m^{2}-1=4m^{2}n+3m^{2}+2m-1$ FLOPs.
